# Density-Dependent Processes in the Life History of Fishes: Evidence from Laboratory Populations of Zebrafish *Danio rerio*

**DOI:** 10.1371/journal.pone.0037550

**Published:** 2012-05-24

**Authors:** Charles R. E. Hazlerigg, Kai Lorenzen, Pernille Thorbek, James R. Wheeler, Charles R. Tyler

**Affiliations:** 1 Division of Biology, Imperial College London, Ascot, United Kingdom; 2 Ecotoxicology and Aquatic Biology Research Group, University of Exeter, Exeter, United Kingdom; 3 Fisheries and Aquatic Sciences, School of Forest Resources and Conservation, University of Florida, Gainesville, Florida, United States of America; 4 Syngenta, Environmental Safety, Jealott's Hill International Research Centre, Bracknell, United Kingdom; University of California San Diego, United States of America

## Abstract

Population regulation is fundamental to the long-term persistence of populations and their responses to harvesting, habitat modification, and exposure to toxic chemicals. In fish and other organisms with complex life histories, regulation may involve density dependence in different life-stages and vital rates. We studied density dependence in body growth and mortality through the life-cycle of laboratory populations of zebrafish *Danio rerio*. When feed input was held constant at population-level (leading to resource limitation), body growth was strongly density-dependent in the late juvenile and adult phases of the life-cycle. Density dependence in mortality was strong during the early juvenile phase but declined thereafter and virtually ceased prior to maturation. Provision of feed in proportion to individual requirements (easing resource limitation) removed density dependence in growth and substantially reduced density dependence in mortality, thus indicating that ‘bottom-up’ effects act on growth as well as mortality, but most strongly on growth. Both growth and mortality played an important role in population regulation, with density-dependent growth having the greater impact on population biomass while mortality had the greatest impact on numbers. We demonstrate a clear ontogenic pattern of change in density-dependent processes within populations of a very small (maximum length 5 mm) fish, maintained in constant homogeneous laboratory conditions. The patterns are consistent with those distilled from studies on wild fish populations, indicating the presence of broad ontogenic patterns in density-dependent processes that are invariant to maximum body size and hold in homogeneous laboratory, as well as complex natural environments.

## Introduction

Fundamental to the long-term persistence of populations is their adjustment and regulation in response to harvesting, habitat modification, and exposure to chemicals [1]–[3]. Regulation is effected through density dependence in life history characteristics (survival, body growth or reproductive output), and may result from intraspecific competition, predation or parasitism which may vary in intensity throughout the life-cycle [4]–[5].

Fish and many aquatic invertebrates have complex life histories with stages that differ greatly in size, morphology, and ecological requirements and which may occupy different habitats. These life-stages also differ in the degree to which they are subject to density dependence and in the life history traits (features of survivorship, growth, development and reproduction) most affected. In fish, density dependence in juvenile mortality has been widely documented and is generally assumed to be the most important mechanism of population regulation [2], [6], 7. For example, Iles & Beverton [8] showed how in plaice, *Pleuronectes platessa*, environmentally induced variability in the abundance of egg and larval life-stages was greatly reduced within a month of settlement, demonstrating the presence of density-dependent survival on the juvenile life-stage. The mechanism for density-dependent mortality is often thought to be through predation, with initial hypotheses developed by Van der Veer [9] and Myers & Cadigan [6] now supported by experimental studies [10]–[11]. However, recent research indicates that density dependence in other life history traits and phases of the life-cycle, in particular density-dependent body growth in juveniles and adults, can be equally important and add substantially to the compensatory reserve of fish populations [2], [12]–[13]. Munch et al. [14] hypothesize that bottom-up processes of regulation (those linked with resource limitation) are likely to act in later phases of the life-cycle while top-down processes (primarily linked to predation and parasitism) are most important at early life-stages. In a further study modelling the population dynamics of coral reef fish, Sandin & Pacala [15] show that while top-down regulation in juveniles suppresses variability in population numbers, bottom-up regulation in adults strongly affects variability in biomass. There is, thus, increasing empirical evidence and theoretical support for the importance of multiple density-dependent processes, acting at different stages of the life-cycle, in the regulation of fish populations.

Understanding and quantifying regulatory processes in different phases of the life-cycle is crucial for assessing the impacts of a wide range of stressors or management actions on population dynamics. This is particularly true for stressors or management actions that affect early life-stages or juveniles and the effects of which may be compensated for, at least in part, by compensatory density dependence in later stages. This includes exposure to chemicals or adverse environmental conditions to which early life-stages may be particularly sensitive, or releases of hatchery fish to enhance or restore fisheries [16].There is consequently a need for more comprehensive studies on the changing forms and strengths (the ontogenic patterns) of density dependence in fish populations and their contribution to overall population regulation.

Empirical and theoretical studies including those discussed above point to a broadly consistent life-cycle pattern of density-dependent processes, despite the fact that they deal with very different study species and environments. We therefore hypothesize that fish populations show a general life-cycle pattern of density dependence that includes density-dependent mortality increasing from the larval life-stage and into the juvenile life-stage followed by a gradual decrease, and density-dependent growth exerting its greatest influence in late juvenile and adult life-stages. We test this hypothesis experimentally by studying density-dependent processes in the life-cycle of zebrafish (*Danio rerio*), a small-bodied fish maintained in simple, uniform laboratory environments. Effects of density on growth, mortality and maturation were measured through the life-cycle, under two different feeding regimes: a constant feed (CF) input leading to resource limitation, and feeding in proportion to individual requirements (IR), easing resource limitation. These feeding regimes allowed analysis of the mechanisms underlying density-dependent growth and survival with respect to food availability and in the absence of predation. Dynamic simulations based on experimental results were used to assess the importance of the different density-dependent processes in population regulation.

## Results

### Overall ontogenic pattern of density dependence

**Figure 1:**
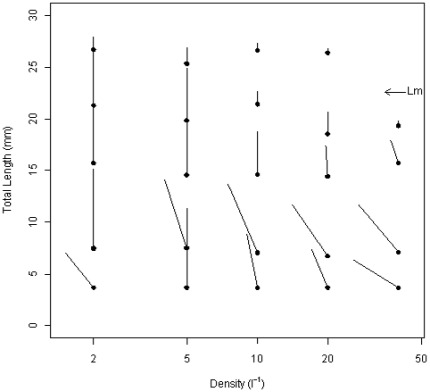
Average total length and density changes of zebrafish in different colonies over 20 day time periods. Starting density and average length for fish in each colony is denoted by the closed circle (•). The final density and average length of the fish for each colony is denoted by the end of the line. The gradient of each line denotes the mortality over 20 days, the shallower the gradient the higher the mortality (note the logarithmic scaled x axis). The vertical length of each line denotes the increase in average length of the zebrafish within colonies; the longer lines (vertically) denote higher individual growth rates. For example, the replicate with a starting density of 2 and a starting average fish body length of 7.4 mm, had a density of 2 (no mortality) and an average fish body length of 15.1 mm after 20 days. Lm denotes the length at maturity determined from the earliest egg production in the experiment.

Short-term experiments maintaining groups of zebrafish at different stages of the life-cycle under a variety of initial density treatments and CF (resource limitation) provide an overview of the ontogeny of density dependence (Fig. 1). In the colonies with fish of a smaller size (starting length under 10 mm), density-dependent mortality gives rise to a sharp reduction in numbers at higher densities, while density dependence in growth is less evident. In the colonies with fish of a larger size (starting lengths at or above 15 mm), there is no evidence of density-dependent mortality but density-dependent growth is clearly present.

### Growth

**Figure 2:**
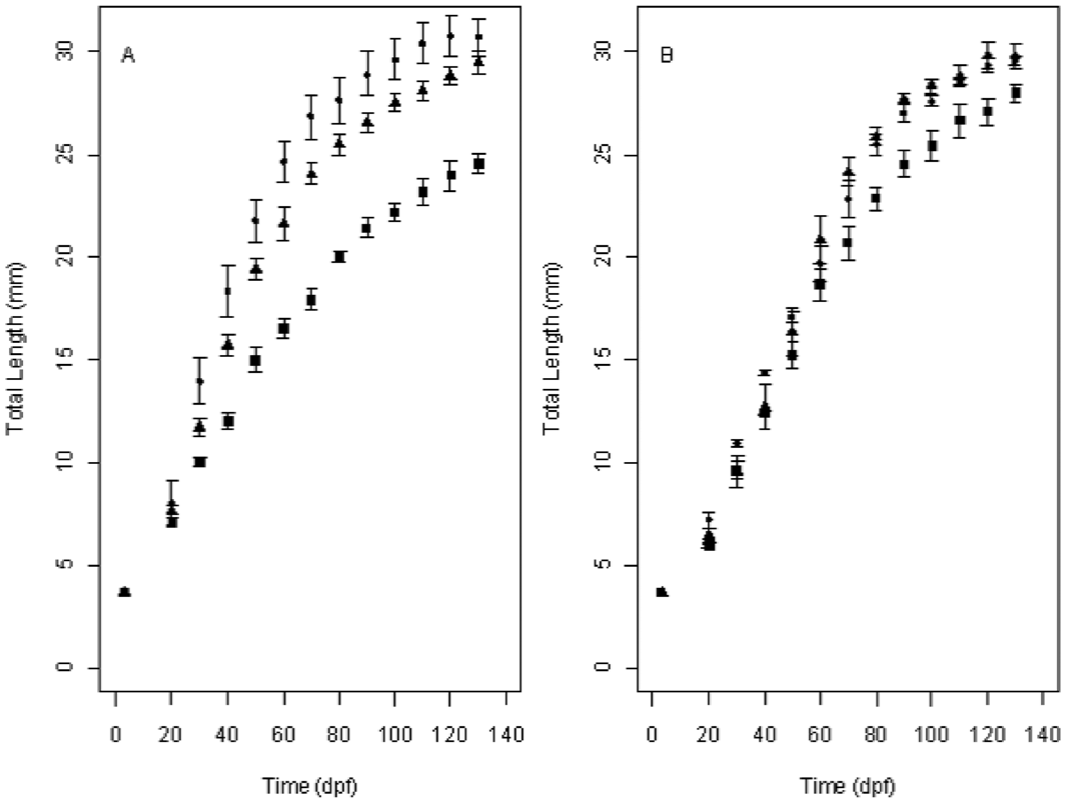
Average total length of zebrafish under two different feed regimes. Average length (±s.e) of zebrafish stocked at three different densities (low density - •, mid density -▴, high density - ▪) over 130 dpf under a constant feed regime (A) and an individual requirement feed regime (B).

Under the CF regime daily growth rates decreased with increasing density (Spearman's (Sp.) Rank −0.435, p = 0.034), but there was no correlation between these factors under the IR regime (Sp. Rank 0.200, p = 0.243). There was strong statistical support for a density-dependent growth model under the CF regime (Akaike weight >0.99), while a density-independent model was supported under the IR regime (Table 1, Fig. 2).

**Table 1:** Comparison of AIC and Akaike weights for the density-independent and the density-dependent body growth models for each feed regime.

	Constant feed		Individual requirement feed	
Growth model	AIC	Akaike weight	AIC	Akaike weight
Density- independent	−113.31	0.002	−72.05	0.614
Density- dependent	−119.48	0.998	−71.12	0.386

### Mortality

**Figure 3:**
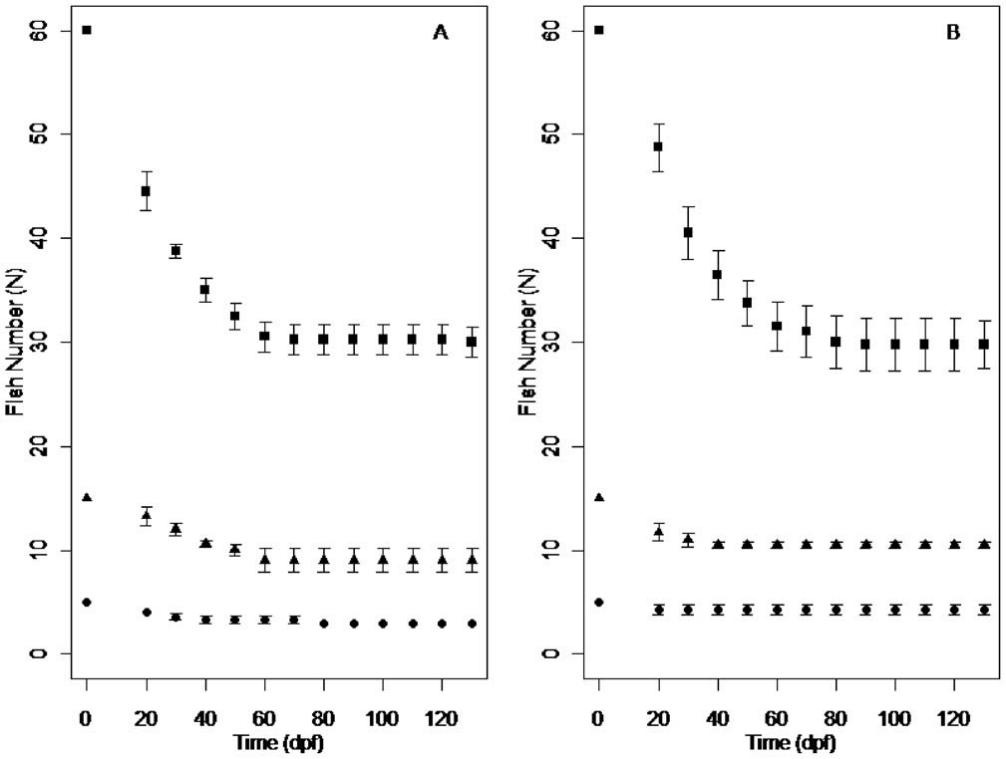
Average number of zebrafish under two different feed regimes. Average number of individual fish (±s.e) stocked at three different densities (low density - •, mid density -▴, high density - ▪) over 130 dpf under a constant feed regime (A) and an individual requirement feed regime (B).

**Figure 4:**
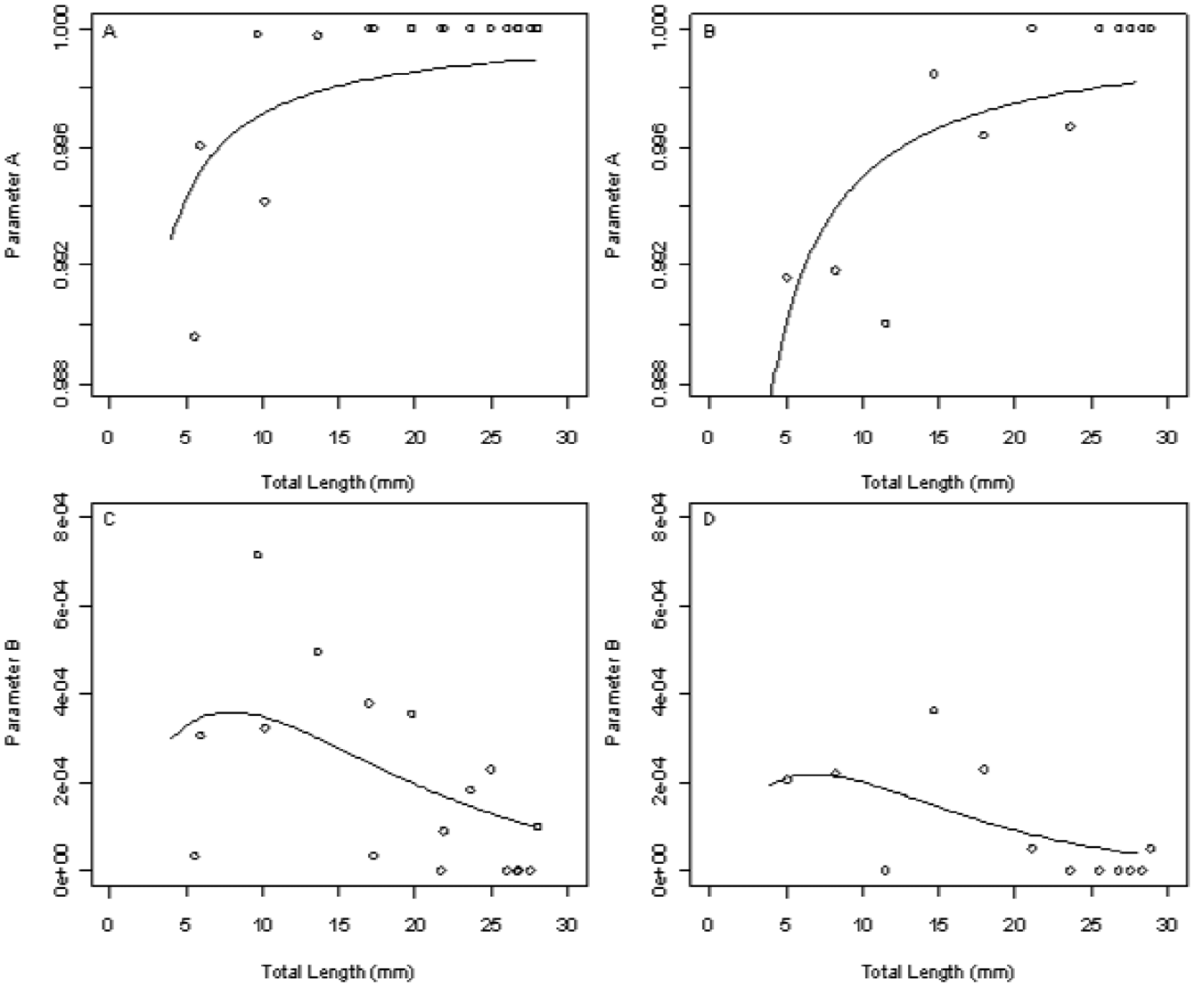
Parameter values for zebrafish survival model under two different feed regimes. Observed (○) and modelled (line) values for parameters *a* and *b* in the Beverton-Holt survival model for constant feed (A & C) and individual requirement feed regimes (B & D). Parameter *A* denotes density-independent mortality and parameter *B* denotes density-dependent mortality.

A time series showing the number of individuals under the CF and IR feed regimes for the long experiment is shown in figure 3. The pattern of density-dependent mortality was described by two parameters: survival at very low density (*a*) and strength of density dependence on survival (*b*). Survival at low density increased with size under both feeding regimes, but more rapidly under CF conditions (Fig. 4). The strength of density dependence (parameter *b*) was highest in fish below about 10 mm length under both feeding regimes (Fig. 4, Table 2). Overall density dependence in mortality was somewhat, but not significantly stronger under CF (resource limited) conditions than under the IR regime. The pattern of change in density dependence with fish size was similar in both treatments, shown by similar *p* values (CF = 0.00036, IR = 0.00022) and *q* values (CF = 7.9, IR = 6.8) (Table 2).

**Table 2:** Parameter estimates (and CIs) for the model relating the strength of density dependence on mortality to fish length (Eqn. 5).

Parameter	Constant feed	Individual requirement feed
Maximum density dependence (*p*)	0.00036 [0.00015, 0.00068]	0.00022 [0.00006, 0.00102]
Length at maximum density dependence (*q*)	7.9 [4.3, 24.2]	6.8 [1.4, 30.6]

### Combined effect of density-dependent processes through the life-cycle

**Figure 5:**
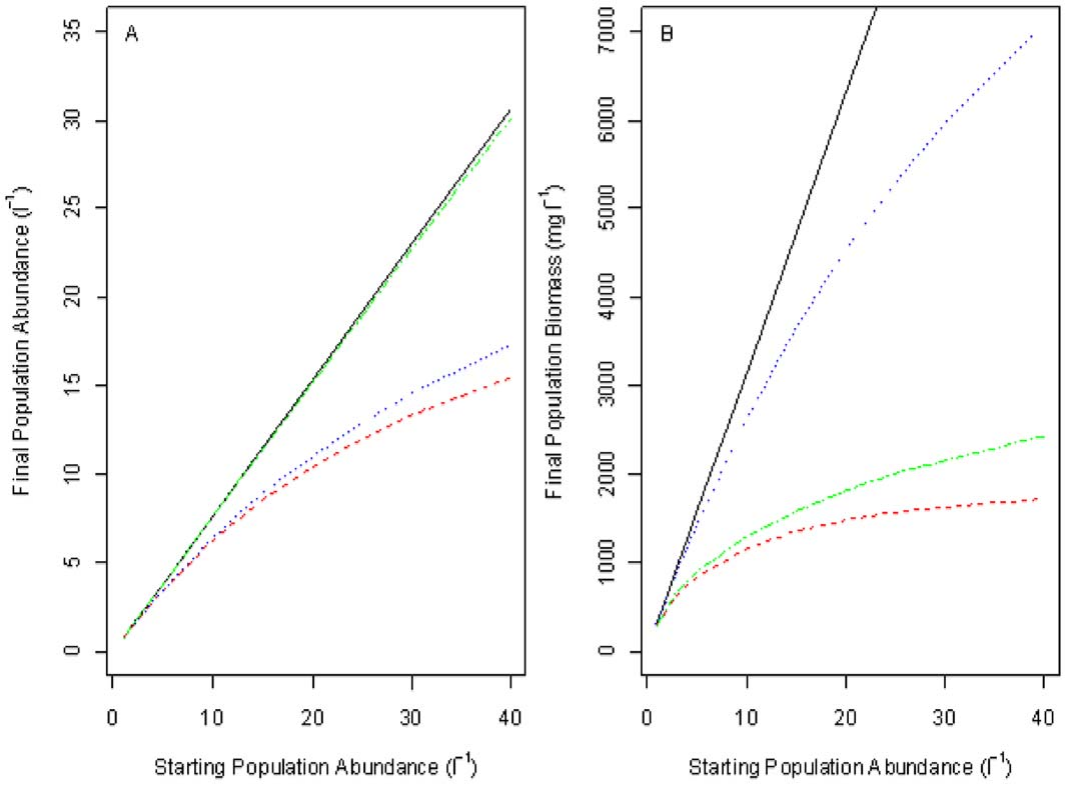
Dynamic simulation results. The results of dynamic simulations into the growth and mortality of zebrafish from hatch to 130 dpf maintained under constant feeding conditions. Final population abundance (A) and final population biomass (B) were calculated under full density dependence (dashed red), density dependence in mortality only (dotted blue), density dependence in growth only (dashed green) and no density dependence (solid black).

A combined difference equation population model with daily time steps including density-dependent mortality and growth functions from the empirical data was formulated for zebrafish between ages 3 and 130 days post fertilisation under the CF regime (i.e. simulated ‘natural’ conditions). The effects of reduced growth rates on maturation and hence reproduction, were not included in this model. The inclusion of density dependence reduced the final population abundance and biomass from that seen for the density-independent version (Fig. 5). Density-dependent mortality had greater effects on population abundance, whilst density-dependent growth had greater effects on population biomass.

### Sex ratio and time to maturation

Sex was clearly determined for all individuals bar one (gonads still in the intermediate development stage). Sex ratio was not significantly different with stocking density under either CF (Anova, F = 0.27, p = 0.76) or IR feeding (Anova, F = 2.5, p = 0.15). Over the duration of the long experiment, only 14 of the 24 zebrafish colonies across both feeding regimes matured and started to reproduce. Whilst there was a trend for delayed maturation in the higher density colonies under the CF regime not reflected under the IR feed regime; the date of first egg production was shown to be independent of colony density under both CF (Anova, F = 4.21, p = 0.104) and IR feeding (Anova, F = 0.64, p = 0.459).

## Discussion

Our study identified a clear ontogenic pattern of density-dependent processes through the life-cycle. Density dependence in mortality was strong during the early juvenile phase but declined thereafter and virtually ceased before fish attained a third of their maximum length. Body growth was strongly density-dependent in the late juvenile and adult phases of the life-cycle. Provision of feed in proportion to individual requirements (easing resource limitation) removed density dependence in growth thus indicating that growth is influenced primarily by ‘bottom-up’ effects. On the other hand, provision of feed in proportion to individual requirements had only a moderate effect on the level of density dependence in mortality, which suggests that juvenile mortality is driven by bottom-up processes to a lesser extent than the growth of larger fish. Both growth and mortality played an important role in population regulation, with density-dependent growth having the greater impact on population biomass while mortality had the greatest impact on numbers.

No significant differences in colony sex ratio or age of first maturation were detected in this study. However, previous work by Lawrence et al. [17] did observe changes in sex ratio due to food availability in the zebrafish, with higher numbers of females developing under higher food availability. We believe that our study did not show consistent effects on sex ratio skew as food became limiting after the period of zebrafish sexual differentiation (35–49 dpf, [18]). Any effects of density or food ration on maturation were also difficult to determine as individual fish could not be tested; only the colony they were in.

The pattern of density dependence within the life-cycle of laboratory maintained zebrafish matches that generally accepted for other, larger, wild fish species, where density dependence in mortality is strongest in juveniles and virtually ceases before fish reach about 25% of their maximum length [7], [19]. Likewise, strong density dependence in growth has been observed in the later stages of the zebrafish life-cycle and this pattern is consistent with that found in larger-bodied, wild species [12]. The presence of a clear ontogenic pattern of density dependence in laboratory populations of a small cyprinid fish with a maximum length of only 5 cm, and its consistency with observations in wild populations of much larger-bodied species (where individuals are subject to changing environmental conditions, unpredictable food supply and predation amongst other differences to our laboratory setup), strongly suggests that this ontogenic pattern of density dependence holds some generality and is invariant to maximum body size.

Food availability has been shown to affect both fish growth through anabolic growth of somatic tissue and provision of energy reserves; as well as survival due to starvation and size-dependent predation resulting from altered growth rates [20]–[21]. Therefore, in periods of low food availability, survival of smaller individuals is reduced due to low energy stores, whilst anabolic growth rather than survival is affected in larger individuals due to higher energy stores. Using two different feed regimes, we investigated the interaction between food availability and the strength of density dependence in the system. The increase in food availability under the IR regime resulted in the loss of density dependence on growth. As this density dependence operates in larger fish, the higher food ration meant these fish had sufficient resources to enable continued growth at all densities. Meanwhile, the strength of density dependence on survival was reduced, but not eliminated. Thus not all individuals were receiving a sufficient ration to maintain their survival. The intra-specific interactions between individuals, potentially through the monopolisation of the food (shown by Hamilton & Dill [22] to occur in zebrafish), could have caused this density dependence on survival to continue, although at a reduced level. We observed the presence of threatening behaviour, such as ‘circling’, which is used as a visual display to assert right to resource access (e.g. food, territory, mating) [23]–[24] and could be one mechanism through which these relationships develop. Another recent study found differences in aggression in zebrafish housed at different densities [25], though whether this played any role in the present study is unclear. Further studies to investigate between behaviour-mediated and energy-mediated causes of density dependence would be possible in order to identify the importance of each mechanism on population regulation Under the CF regime the strength of density dependence on survival is a stronger version of this with more individuals not receiving their resource requirements. Therefore, we suggest that internal changes resulting in increased fat reserves reduce density-dependent mortality in these scenarios. Determining the underlying ontogenetic control on this process would be of further interest in the field of population ecology.

Density-dependent mortality in the early juvenile life-stage is considered the primary force of population regulation in conventional fisheries models [12], [26] and it is generally accepted that the mechanism for this is through predation [2]. In some systems, the mechanisms of density-dependent mortality have been elucidated in detail, for example in coral reef fish where space competition and predation for species that ‘exhibit’ are the primary causes of density-dependent mortality [27]–[28]. Our result that in the absence of top-down processes (primarily predation), population regulation through density-dependent mortality in juveniles is still apparent means that bottom-up processes have a role not only in effects on growth in later life-stages, but also on survival at earlier life-stages. It also suggests that bottom-up drivers on the compensatory response of populations may be under-appreciated. A better understanding of the role of bottom-up processes would enhance our understanding of population-level responses to exploitation and stress.

Our results have important implications for the study of population dynamics. They indicate a general pattern of density dependence in fish life histories that can inform the structure of population models and aid targeting of field studies in population regulation on those aspects of the most crucial life-stages and processes. This is particularly relevant to stressors or management actions that affect early life-stages or juveniles such as exposure to chemicals or adverse environmental conditions to which early life-stages may be particularly sensitive, or releases of hatchery fish to enhance or restore fisheries [16]. The primary protection goal of risk assessment is the persistence of functioning populations and their ecosystem services [29]. As current risk assessment practices collect data at the individual-level in the laboratory, extrapolation of these results to the population-level is needed and population models can be used to address this issue [30]–[31]. Compensatory density dependence defines the limits to which populations can persist when subjected to additional sources of mortality and is therefore an essential aspect of these population models [32]–[33]. In fisheries enhancements, where hatchery-reared juveniles are released into natural populations, compensatory density dependence defines the limits to which such releases can increase overall population abundance and the degree to which hatchery fish will displace wild fish through competitive interactions [16]. Quantifying the lifetime pattern of density-dependent processes is crucial to predicting outcomes of fisheries enhancements releasing fish at different life-stages.

These studies on the zebrafish further support the use of this organism for studying population-level processes in fish. Whilst the zebrafish is small and not of interest directly to fisheries research or conservation, many of the ecological processes in its life-cycle are similar to those that are and hence it can also be used as a study species to learn more about general ecological patterns. It has already been widely used in many other research areas due to its species specific attributes, including its small size, ease of culture and short generation time [34]–[35]. The species is now also regularly used in behavioural studies [23]–[24], [36]–[37] and the effect of behavioural development on density-dependent mortality is something that has yet to be investigated, but could result in important alterations to the strength of density dependence. Caution must be exercised, however, when extrapolating from laboratory studies to wild populations because laboratory animals are subject to domestication effects resulting from natural and artificial selection and from developmental responses to the laboratory environment, that are known to affect many aspects of the organism's biology and life history [38].

### Conclusions

Our study demonstrates clear patterns of ontogenic change in regulatory processes with populations of laboratory maintained zebrafish. The patterns are consistent with those distilled from literature studies on wild fish populations, indicating the presence of broad ontogenic patterns in density-dependent processes that are invariant to maximum body size and hold in homogeneous laboratory, as well as complex natural environments.

## Materials and Methods

### Experiments

Experiments were conducted between December 2008 and April 2009 in the University of Exeter aquarium facilities. Wild Indian Karyotype (WIK) strain zebrafish embryos were collected from a stock colony and divided into two groups for the short-term and long-term experiments.

### Short-term experiments

Zebrafish embryos were housed in a 30 L stock tank. Every 22 days until 91 days post fertilisation (dpf), 154 zebrafish were removed from the stock tank and individuals measured for total body length (from the most forward point of the head to the tip of the tail, from here-on called length) using a digital camera (Nikon SMZ1500, Nikon Ltd, Japan) where the fish was placed on a measurement grid background. ImageJ (National Institutes of Health, U.S) was used to accurately measure the fish length. Fish were then transferred randomly into one of five 2 L tanks at densities 4, 10, 20, 40 and 80 fish per tank. Each colony was fed a constant ration (CF) of 40 mg of dry feed daily (Food products: ZM000, ZM100, ZM200 and ZM300, Zebrafish Management Ltd, UK). This feed level resulted in fish stocked at low densities having access to >5% body weight of feed per day throughout the experiment, whilst fish stocked at high densities had <1% body weight of feed per day by the end of the experiment. As current zebrafish husbandry standards suggest 3–8% of body weight of feed per day [39], the feed regimes in this experiment resulted in resource limitation at higher fish lengths and population densities, allowing the effects of bottom-up resource limitation on density dependence relationships within these colonies to be investigated. After 20 days in those tanks, individual length was recorded and survival rate determined. These fish were subsequently transferred to a separate stock tank and not used further in these experiments.

### Long-term experiment

Zebrafish embryos were collected and transferred into 2 L tanks at densities 5, 15 and 60 fish per tank, with eight tanks for each density in a random block design for 130 days. Two different feeding regimes were used. Half of the colonies were fed a constant ration (CF) of 50 mg of dry feed daily where resource becomes progressively limiting with increasing fish size and population density; whilst the other half under the individual requirement (IR) feed were fed dependent upon the average fish size and number of fish in their tank colony (Eqn. 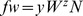, where *y* = 0.1932, *z* = 0.75, W is zebrafish average wet weight and N is number of zebrafish in the colony), where ration is adjusted progressively during life with fish growth to meet required energy demands. The individual feed requirement for each colony under this feed regime was recalculated each sample day (every 10 days), using the new data for weight and survival. Every 10 days, individual fish were measured for length (as above) and survival data were collected. From day 70 post fertilisation, spawning chambers were introduced to each tank to assess for daily egg production.

The fish in this experiment were sacrificed after 130 days and the body was fixed in Bouins Solution (Raymond A. Lamb Ltd, Eastbourne, UK) for 24 hours then stored in 70% industrial methylated spirits (IMS, Fisher Scientific, Loughborough, UK). Subsequently, fish bodies were dehydrated and embedded in paraffin wax (Sigma-Aldrich, Gillingham, UK) using a Shandon tissue processor (Thermo Electron Corporation, Runcorn, UK). Each fish was sectioned, mounted on glass slides and stained with haematoxylin and eosin (ThermoShandon, Pittsburgh, US) and treated with Histomount (National Diagnostics, Hull, UK). Sex was determined using light microscopy to identify male or female gametogenesis.

Tank flow rates were set dependent on the number of individuals in the tank colony; with each individual adding 0.05 l hr^−1^ to the overall total (e.g. 60 individuals in the tank had a water flow rate of 3 l hr^−1^). These flow rates were set deliberately high to maintain good water quality throughout the experiment. Additionally, water temperature was maintained at 26±1.5°C, dissolved oxygen >60% and pH 6.5±0.5. Photoperiod was on a 12∶12 light∶dark cycle.

### Statistical Analysis

The purpose of the statistical analyses was to address two different questions. The first was to determine the ontogenic progression of density dependence relationships across the life-cycle of the zebrafish. The second was to determine the strength and form of these density-dependent relationships for growth and survival when subject to resource limited and resource abundant scenarios. In order to fit the growth and survival models below, the data from both experiments were combined. Specifically, for the growth models (Eqns. 1 & 2), the models were fitted to data-points for biomass and growth rate taken from both experiments. For the mortality sub-models (Eqns. 4 & 5), the models of *a(L)* and *b(L)* were fitted to data-points for numbers and survival rate taken from both experiments. Anova and Spearmans rank analyses were used to investigate general trends between growth rate, sex ratio and date of initial spawning with density (Minitab 15, Minitab Inc., USA). Two different growth functions were fitted separately to the CF and IR feeding regimes: a density-independent von Bertalanffy Growth Function [40]

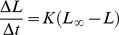
(1)and a density-dependent version [41]

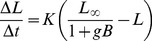
(2)where *L_∞_* is the asymptotic length, *L* is current length, *K* is a growth rate, *B* is biomass and *g* is the density-dependent parameter. These models were confronted with data using model selection based on the Akaike Information Criterion (AIC, method see Hobbs & Hilborn [42]) to identify the best model fit to the data.

Within each inter-sample period, density dependence on survival was expressed by the Beverton-Holt model [43]

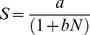
(3)where *a* is survival at very low abundance (N = 0), *b* is a density-dependent mortality constant and *N* is the population abundance. The relationships between survival model parameters and fish size were then examined graphically and by fitting models that describe the parameters *a* and *b* as functions of length (Eqns. 4 & 5).
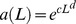
(4)

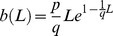
(5)Equation (4) describes the size dependence of survival at very low abundance by means of an allometric relationship between mortality and length, with exponent *d* and mortality at unit length *c*. Equation (5) describes the relationship between the strength of density dependence on survival in fish by means of a dome-shaped exponential function with maximum *p* and length-at-maximum *q*. Likelihood profiles were computed in Excel (Microsoft, USA) and confidence limits were determined from these profiles [44].

### Dynamic simulation of density dependence

The purpose of this dynamic simulation was to determine the importance of density-dependent growth and survival on a population's recruitment and biomass. The parameterised models from the empirical data for growth and survival were used to simulate the final abundance and biomass within the system when starting with a range of initial abundances between 1 and 40 fish larvae. The simulations were scaled on a 1 L tank and followed a zebrafish population in the system between 3 and 130 dpf. Fish introduced into the system at 3 dpf were 3 mm in length and 0.16 mg in weight. Each day the fish increased in length dependent upon the parameterised growth model (Eqns. 1 & 2) and fish survival was dependent upon the parameterised survival model (Eqns. 4 & 5). For each starting abundance four different simulations were conducted: 1) Density-dependent growth and survival, 2) Density-independent growth and survival, 3) Density-dependent growth and density-independent survival and 4) Density-independent growth and density-dependent survival. The simulations enabled the density-dependent growth and survival to be turned on and off (impossible within the initial experimental setup) and allowed conclusions to be drawn as to the importance of these processes on population recruitment and biomass. Fish weight was calculated from body length according to the classic allometric function: 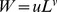 where *u* = 0.0048 and *v* = 3.1988. Fish reproduction was not included in these simulations.
